# A Review on Technical and Clinical Impact of Microsoft Kinect on Physical Therapy and Rehabilitation

**DOI:** 10.1155/2014/846514

**Published:** 2014-12-10

**Authors:** Hossein Mousavi Hondori, Maryam Khademi

**Affiliations:** ^1^Department of Neurology, School of Medicine, University of California, Irvine, CA 92617, USA; ^2^School of Information and Computer Science, University of California, Irvine, CA 92617, USA

## Abstract

This paper reviews technical and clinical impact of the Microsoft Kinect in physical therapy and rehabilitation. It covers the studies on patients with neurological disorders including stroke, Parkinson's, cerebral palsy, and MS as well as the elderly patients. Search results in Pubmed and Google scholar reveal increasing interest in using Kinect in medical application. Relevant papers are reviewed and divided into three groups: (1) papers which evaluated Kinect's accuracy and reliability, (2) papers which used Kinect for a rehabilitation system and provided clinical evaluation involving patients, and (3) papers which proposed a Kinect-based system for rehabilitation but fell short of providing clinical validation. At last, to serve as technical comparison to help future rehabilitation design other sensors similar to Kinect are reviewed.

## 1. Introduction

Traditionally, a great portion of physical therapy and rehabilitation assessment of stroke patients is based on a therapist's observation and judgment. The assessments methods (e.g., Fugl-Meyer et al. Assessment of Physical Performance [1]) rely heavily on the therapists visual assessment of how the patient is performing a standard task. This process needs a trained Physical Therapist (PT) or Occupational Therapist (OCT) to spend one on one time with the patient. Yet, the assessment can be inaccurate for several reasons one of which is the subjectivity of these behavioral and clinical assessments. Sensor and computing technology that can be used for motion capture have advanced drastically in the past few years; they have become more capable and affordable. Motion capture systems (MoCap) record human body's kinematic data with high accuracy and reliability; analysis of MoCap data results in better clinical and behavioral assessment and more efficient therapeutic decision making accordingly.

The two main families of sensors which have been commonly used in human motion capture for rehabilitation engineering are optoelectronics and nonoptoelectronics sensors [2, 3]. The first groups may or may not use markers to track movements. If they use, markers are attached to the body to represent major skeletal segments and joints while the optical system (a camera and postprocessing vision system) tracks the markers and obtains the body segments and joints' position and orientation. In markerless systems, the image features such as colors, edges, shapes, and/or depth are used to interpret the motions. The nonoptoelectronics sensors include inertial, mechanical, and magnetic systems. In the remainder of this section, we detail the major motion capture sensors which are categorized in Table 1.

The nonvision systems usually use one or a set of sensor(s) to track human motion. For example Wii Remote is a commercial system which uses inertial and optical sensors to measure human motion. Wii is originally designed for interacting with and controlling Nintendo Wii's console but its ability to measure human motion in real time and availability of Software Development Kit (SDK) allowed Rehabilitation Engineering (RE) development (e.g., [4]).

Inertial systems for motion capture use miniature inertial sensors, sensor fusion algorithms, and human skeletal models. Inertial sensors are sometimes combined with other means of motion capture [4] and are used in rehabilitation [5].

Magnetic systems for motion capture consist of a transmitter and receiver(s). Such a system calculates position and orientation of each receiver by measuring the relative magnetic flux of 3 orthogonal coils on both the receiver and transmitter [6]. The sensor's output is 6 DoF per receiver providing 3D position and orientation. Magnetic sensors are very sensitive to presence of magnetic materials; although occlusion is not a problem with nonmagnetic materials, vicinity of most metals used in desks and furniture can compromise the measurement accuracy.

Wearable technology may or may not use the aforementioned sensors (magnetic, inertial, or optical). There is a considerable literature on using wearable sensors in RE. Music Glove [7] and Smart Suite [8] are two examples of wearable sensors that measure hand and full body movement, respectively. Wearable devices sometimes overlap with mechanical systems (i.e., exoskeleton systems). An exoskeleton is usually a rigid mechanical device that the user puts on his/her upper or lower limb [9, 10]. The device can follow the user's motion passively (i.e., just measuring the motion) or actively (i.e., assisting with or resisting against the user's intended motion).

Vision based methods may track movements using either contrast-based or depth-based imaging. Color sensing system in RE may track a specific color marker attached to the patient's body or held by their hand [11] or track the patient's skin color [12]. On the other hand, systems which use depth imaging [13] may use the skeletal tracking that devices such as Microsoft Kinect or Leap Motion Sensor provide. Depth-based systems may also use depth segmentation and computer vision algorithms to detect and track human body from the sequences of depth images [14].

This paper mainly reviews the notable contrast-based MoCap systems used in rehabilitation with more emphasis on depth imaging technologies (i.e., Microsoft Kinect). In Section 2, we provide details on how we searched for papers and give statistics on how many papers are published each year on this subject matter.  Section 3 talks about RE systems before the release of the Kinect to the marker; the sections try to contrast the impact of Kinect on the field and showed how far systems could go before Kinect was available.  Section 4 reviews technical considerations that developers and clinicians need to consider for using Kinect in their RE systems; it reviews papers which examined Kinect's reliability and accuracy. In Section 5, we discuss a wide range of systems which used Kinect for rehabilitation: Section 5.1 goes through the studies with clinical evaluation and Section 5.2 reviews those without clear clinical evaluation.  Section 6 reviews Kinect-like sensors, provides their specs in comparison with Kinect, and names a few examples of RE studies which used these sensors.

## 2. Search Methodology and Statistics

**Figure 1:**
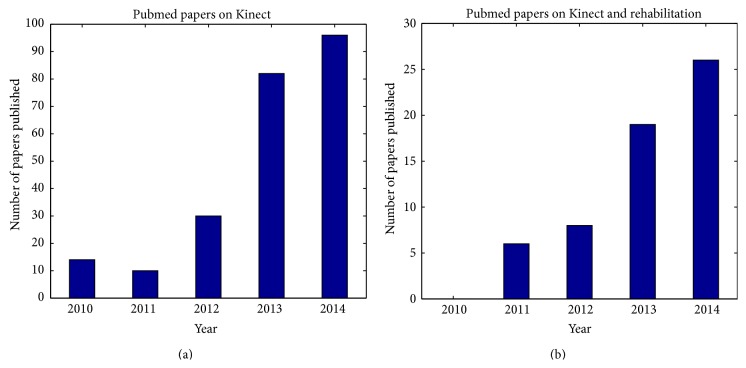
Annual publications of papers on Kinect indexed by Pubmed; paper that mentioned Kinect (a) and papers that mentioned Kinect and rehabilitation are (b).

**Figure 2:**
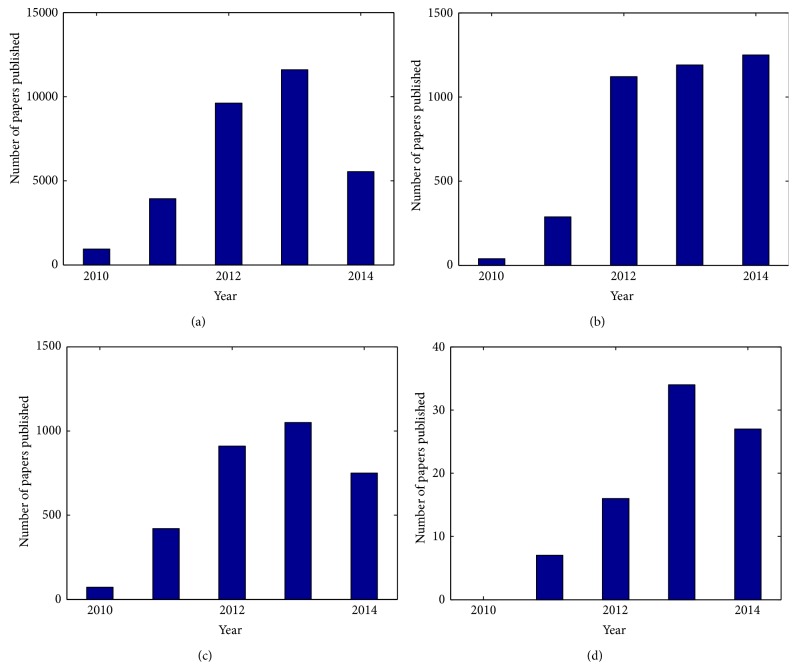
Annual publications of papers on Kinect indexed by Google Scholar. (a) and (b) show the number of papers that mention “Kinect” and “Kinect + Rehabilitation”, respectively. (c) and (d) show the number of papers that mention “Kinect” and “Kinect + Rehabilitation” in the title, respectively.

Figure 1 shows the search results for papers on Kinect and rehabilitation published in Pubmed from 2010, advent of Kinect, to October 2014 when this review paper is being prepared. Also, a search on Google scholar shows that from 2010 till now 18500 papers (which are indexed on Google scholar) have mentioned “Kinect” of which 3240 have also mentioned “rehabilitation”. Since not every referral to these words means that the topic of the article is on Kinect and/or rehabilitation, we applied two filters to limit the results to the papers which mention Kinect and/or rehabilitation in the title.  Figure 2 shows the search results with further details; the growth of the published papers in RE field which used Kinect shows that the technology has appeared promising to RE developers and clinicians.

## 3. Rehabilitation Systems before the Advent of Kinect

This section reviews some of the RE studies which became a paradigm for MoCap-based RE systems and greatly affected how RE developers adopted and used the Kinect; in most of these systems a home-based MoCap system using a digital camera is proposed which is used for controlling a rehab game.

Conventional digital imaging, or RGB imaging, is done using the ubiquitous digital cameras which record color images with standard properties. RGB image processing has been used in many RE cases to achieve motion capture. The reason is that validity of the RGB MoCap system is easily done by visual investigation; that is, one can confirm, via visual observation, if the system is actually tracking the color marker or subject's movement in the raw and processed RGB video.

In this section, we discuss Sucar et al. [12] as a systematically complete rehabilitation system with clinical evaluation which made use of digital imaging for motion capture. Other papers with similar motion capture methods are then summarized and briefly discussed. They developed a low cost computer vision system that tracked the stroke patient's hand and obtained its 3D position using two perpendicular cameras. They used this 3D position as an input to play a web-based virtual reality (VR) game. Introducing games facilitated the repetitive movement by engaging the patient [15]. The system [16] required calibration and used color segmentation to detect skin color [17].

Two main limitations of such a system are as follows: (1) it can confuse human hand with objects that have a similar color as human skin (e.g., wooden surfaces) and (2) the patient has to wear long sleeves to cover the forearm skin; otherwise it is mistakenly detected by the system and the center of the detected area (hand + forearm) will not be the center of hand anymore.

The authors conducted a pilot study with 11 stroke patients of whom 5 used the Gesture Therapy system and 6 underwent conventional therapy (control group). The patients who used Gesture Therapy used it for 6 sessions of 20–45 minutes. The second group received traditional therapy with similar intensity. Both groups showed significant improvements in their FM scores (*P* < 0.005). They observed no significant difference between the FM scores of the 2 groups which shows that the VR therapy was as effective as the conventional treatment.

In another work using a dual camera markerless motion capture system, Evett et al. developed a platform for stroke rehabilitation [18]. Their system leveraged a webcam and a thermal camera to recognize hand gestures. The limitations of this system are that it requires training to recognize gestures and then it only recognized two gestures (hand open and closed).

Pridmore et al. [19] developed a Mixed Reality (MR) environment for stroke rehabilitation. MR is an interface between the real and virtual environments [11]. MR allows the patient to have more sense of realism than virtual reality environment. The learned (or relearned) skills in MR may generalize more to corresponding real-world situations (compared with virtual reality). Another platform for vision based games for poststroke rehabilitation is developed by Burke et al. in [20]. Their system offered two games which are controlled by motion capture via color/object segmentation and motion detection. These systems are typical examples of motion capture systems based on color tracking with little or no clinical evaluation.

The common shortcoming in most of the aforementioned systems is that they do not use spatial calibration techniques (except [12, 21, 22]). Instead, they rely on the pixelwise position of the subject's detected hand in the image space which means that distance between the user and the camera affects the measurements [23]. Tao and Hu [24] evaluated their color segmentation motion capture method by comparing it with the result simultaneously obtained from a commercial infrared (IR) marker-based tracking system. Such commercial systems use IR cameras and obtain the 3D position of IR reflective markers or IR LED markers very accurately. However, due to considerable initial cost and costly maintenance they are not applicable in home-based rehabilitation. Apart from financial costs, other limitations of such systems are the necessity for attaching many markers to the body and cumbersome installation and calibration. In spite of these limitations, these systems are used to give a ground truth for calibration of a color-based marker tracking systems.

## 4. Kinect Sensor: Technical Considerations for RE Development

Compared with an RGB image, a depth image contains information relating to the distance of the 3D objects surfaces from the camera. Depth image reveals extra information about 3D position of pixels. Extracting depth information from an RGB image is not trivial and is computationally expensive. Also, with depth information, segmentation and background subtraction becomes considerably easier and more accurate; this encourages RE developers to prefer depth sensors over RGB cameras in motion capture applications. An important feature of the available depth sensors is skeleton tracking. For example, Microsoft Kinect provides a Software Development Kit (SDK) which gives developers access to body joint positions and orientations.

### 4.1. Kincet's Specifications

Kinect was originally built for Xbox 360 video game console, but later the Windows PC version of it was also released. Kinect provides users with a Natural User Interface (NUI); users can control the system/game using either gesture or voice commands. The software and camera technology of Kinect were developed separately by Rare and Prime Sense, respectively. The original Prime Sense's system was composed of an infrared projector, a camera, and a microchip. It was able to track 3D objects in the real world.

Kinect (i.e., original Kinect model) is a 14.8′′ × 5.9′′ × 4.8′′ bar (3.1 pounds) that is usually placed below/above the screen. It consists of an RGB camera, a depth sensor, and a multiarray microphone. The added value of Kinect compared with other cameras is its depth sensor that offers capturing 3D data independent of lighting conditions. To accomplish this, the depth sensor has an infrared projector and a monochrome CMOS sensor.

The Kinect sensor can capture RGB, depth, and infrared streams with frame rate of 9–30 Hz based on resolution. The default display resolution of these streams is 640 × 480 pixels, but it can be increased up to 1280 × 1024 with a lower frame rate. The RGB stream comes in 8 bit resolution in either color format of VGA or UYVY whereas the depth stream is 11 bit allowing for 2048 different depth sensitivity levels.

Kinect's depth sensor can be adjusted to either near (seated) or far (default) range mode. In seated mode, people within the range of 0.4–3 m (1.3–9.8 ft) can be seen, though the recommended practical range of this mode is 0.8–2.5 m (2.6–8.2 ft). In default mode, standing people within 0.8–4 m (2.6–13.1 ft) are detectable. The recommended practical range of this mode is 1.2–3.5 m (3.9–11.5 ft). Its angular field of view is 57° horizontal and 43° vertical with a pivot able to tilt 27° up or down. Kinect's microphone can process 4 channels of 16-bit audio at a sampling rate of 16 kHz. The Kinect sensor can recognize 6 people but only track 2 of them. Developers can access the Kinect's raw data from any of depth, RGB streams, or microphone array. They can benefit from skeletal information (including 20 joints per active individual) of up to 2 from 6 recognized individuals. Only individuals who face the sensor can be recognized. For development purposes, there is a SDK for Windows that can be programmed in different languages of C++, C#, and Visual Basic.Net (dot Net).

### 4.2. Evaluation of Kincet's Accuracy and Reliability

Motion capture using Kinect has become increasingly popular in physical therapy and rehabilitation; hence understanding limitations of the Kinect sensor is important. In the current section, we discuss the work centered around evaluating Kinect as a robust and reliable sensor.

There have been several attempts to evaluate the Kinect's measurements quantitatively. Obdrzalek et al. [25], Mobini et al. [26], and Fernández-Baena et al. [27] investigated the accuracy of Kinect's joint tracking, specifically whether Kinect's joint localization and pose estimation is robust and reliable. For example, Obdrzalek et al. [25] specified 6 physical exercises to examine pose accuracy of Kinect. The results show that Kinect is a good option to be used as an online motion capture device because of its low price. However, the Kinect skeleton tracking suffers from occlusion or having objects such as chairs in the scene (the chair's leg can be detected as an individual's leg in the seated mode). Therefore, developers should consider addressing issues such as occlusion, self-occlusion, and unconventional body postures or use of wheelchair/walkers. Mobini et al. [26] first used a fabricated model of the upper body. Then, they estimated the displacement between various joints by Kinect and compared them with the actual values from the model. Fernández-Baena et al. [27] compared precision in the computation of joint angles between Kinect and Vicon which is one of the commercial IR trigonometry MoCap systems. Based on the obtained results, Kinect is precise enough for most of the clinical rehabilitation treatments. In a similar attempt, Dutta [28] compared Kinect with Vicon to investigate whether Kinect is sensitive enough to be used as a 3D MoCap device. Stone and Skubic [29] validated Kinect's ability in the elderly fall monitoring and compared it against Vicon measurements and showed that Kinect provides acceptable accuracy.

Kurillo et al. [30] examined Kinect's accuracy by measuring the upper extremity's reachable workspace of 10 healthy individuals; movements were recorded using Kinect and an IR marker-based motion capture system simultaneously. The results showed that the Kinect-based system provides sufficient accuracy and reliable results compared to the MoCap system.

Bonnechère et al. [29, 31, 32] validated range of motion (ROM) measurements using the Kinect with concurrent measurement performed by traditional marker-based stereophotogrammetry system; 48 volunteers were asked to perform shoulder abduction, elbow flexion, hip abduction, and knee flexion motions in 2 sessions. Kinect and the maker-based system shows similar statistical trends in the recorded data but in some cases the measured ROMs were different. van Diest et al. [33] evaluated Kinect's suitability for movement tracking in exergaming in 20 healthy individuals. They showed that both Kinect and Vicon capture more than 90% variance of all body segment movements within 3 principal components. They showed that Kinect tracks trunks movement accurately but it may underestimate arm movements and overestimate leg movements by up to 30%. Clark et el. [34, 35] showed that through calibration with the 3D MoCap system, Kinect yields a significantly better accuracy.

Pfister et al. [36] showed that Kinect has basic MoCap capabilities but requires minor adjustments to be an acceptable tool for gait monitoring. Hawi et al. [37] showed that Kinect provides good test-retest reliability, but lower accuracy versus goniometer measurements. Antón et al. [38] proposed a computational algorithm to improve Kinect's motion tracking accuracy. Bo et al. [39] used Kalman filtering and inertial measurement (sensor fusion [40]) to reduce tracking errors of Kinect and evaluated their method with healthy individuals.

Tanaka et al. [41] and Taylor et al. [42] explored the possibility of using different game console interfaces for rehabilitation programs. For example, Tanaka et al. [41] chose Sony PlayStation Move, Nintendo Wii, and Microsoft Xbox 360 Kinect to compare them in terms of specification, required therapeutic motion, and motion captured; their results address the research implications of using these interfaces. Mortensen et al. [43] used various video gaming consoles for rehabilitation of 15 females with fibromyalgia syndrome (FMS); the patients reported their intervention as a helpful distraction from their chronic pain and reported that the Kinect was their preferred console compared with Nintendo Wii and PlayStation 3 Move.

The overall lesson from these studies is that Kinect is an acceptable and affordable depth sensor for rehabilitation purposes. But developers should take note of problems with occlusions in the image and noises in skeleton tracking. To solve these problems, use of Kalman filter, sensor fusion, and calibration were proposed. To help the transition from a commercial gaming tool to a therapeutic tool, Levac et al. [44] proposed a knowledge translation (KT) recourse to improve decision making in the clinical use of the Kinect.

## 5. Clinical Evaluation of Kinect

The advent of affordable depth imaging technology has made an enormous impact on motion capture in rehabilitation; ever since Microsoft Kinect has become available to developers, many papers have been published on rehabilitation using the Kinect sensor. The current section discusses the developed systems using Kinect that targeted assessment of poststroke physical disability and rehabilitation. It separates the clinically evaluated systems from the systems with no evidence of clinical experiments and reviews both.

### 5.1. Systems Evaluated by Patients with Neurological Disorders

Table 2 summarizes the Kinect-based studies with clinical evaluation. Lloréns et el. [45] created a game to promote walking rehabilitation using Kinect as the motion capture controller. In their game, the patient stands in front of a screen while his/her movements are monitored using Kinect. A pair of virtual shoes is displayed on the monitor following the patient's feet movements; the shoes are a partial avatar of the patient in a nonimmersive virtual reality environment. In the game, the patient had to move his/her feet so that the virtual shoes move and step on some virtual objects that appeared on the screen while avoiding some other virtual objects. 15 chronic patients (6 months to 17 years after stroke) participated in this study for lower limb rehabilitation. Each patient played the game for 20 sessions during 3–5 weeks; each session was 45 minutes. Then, Wiederhold and Riva [46] conducted a within subject analysis; they ran two clinical tests of balance before the initial session and after the final session. The result showed significant (*P* < 0.01) improvement in balance recovery as measured by the Berg Balance Scale (or BBS) but not significant (*P* = 0.08) difference in Performance Oriented Mobility Assessment (or POMA or Tinetti test).

Chang et al. [47] used Kinect for vocational rehabilitation in preservice training to increase professional independence and accelerate community integration. Four subjects with cognitive impairments participated in the training for 2 weeks; the authors recorded the patients' feedback qualitatively using a Likert scale. The authors showed the system was helpful for preservice training but they did not use any clinical scales.

Exell et al. [48] used Kinect's skeletal tracking for an upper limb rehabilitation. They used functional electrical stimulation (FES) to facilitate and rehabilitate patient's arm movement while the patient was also being assisted by a weight-compensating mechanical system. The subject interacted with objects by picking them up and placing them in a directed position. Exell et al. evaluated their system with 1 stroke patient. The patient played the game for 18 sessions and they showed that the mean joint angle error across the 3 main upper extremity joints reduced between 35 and 51%.

Gama et al. [49] and Pastor et al. [50] proposed two simple systems for poststroke upper limb rehabilitation. They evaluated their systems with one patient. Gama et al. concluded that Kinect is accurate enough and suggested that further potential studies can be done.

Acosta [51] also developed a system using Kinect for upper limb rehabilitation. For evaluation, he recruited 11 subjects. Six patients and 5 able-bodied individuals (control group) participated, using the system for 6–10 days. The patients' upper extremity Fugl-Meyer scores before and after the program were obtained. All patients maintained or improved their FM scores but the change was not significant. This study showed that therapy using the Kinect-based system maintained or improved the patients' motor performance. Adams et al. [52] recruited 14 hemiparetic stroke patients who received Virtual Occupational Therapy Assistant (VOTA). Spearman's rank correlation analysis indicates a moderate correlation between VOTA-derived metrics and the time-based WMFT assessments, supporting the criterion validity of VOTA measures as a means of tracking patient progress during an UE rehabilitation program that includes practice of virtual ADLs.

Lee [53] showed that among 14 patients with stroke those who received Kinect-based versus those in conventional treatment demonstrated more clinical and behavioral measures with significant improvements after the treatment (5 significantly improved measures in the experimental group versus 2 in control group). Sin and Lee [54] showed Kinect-based rehabilitation treatment on top of conventional treatment yields significantly greater improvements in functional and behavioral measures (FMA and BBT) as validated in a group of 20 patients with stroke compared with a group of 20 control patients.

One of the notable studies involving the Kinect for rehabilitation is Bao et al. [55]. Five patients with stroke improved their Fugl-Meyer and Wolf Motor Function Test scores and after 3 weeks of training retained some levels of improvements in 12-week follow-up. Using fMRI, they showed that in patients with stroke the neurological underpinning of learning a task using Kinect may differ from healthy control subjects. Based on the aforementioned studies on patients with stroke, Kinect has demonstrated great potentials in rehabilitation and assessment of patients with stroke.

Apart from stroke, Kinect has been proposed for similar rehabilitation, assessment, and monitoring systems; for example, the following studies targeted elderly care and used Kinect in their systems. Dutta et al. [56] recruited 10 older adults, extracted balance data using Kinect and Wii, and showed that the maximum Center-of-Mass (CoM)-Center-of-Pressure (CoP) lean-angle correlates significantly with the clinical balance scores (i.e., Berg Balance Scale). Pu et al. [57] investigated key factors affecting the balance in older adults using Kinect. They recruited older adults and showed that the static and dynamic balance functions were related with distinct factors. Hsieh et al. [58] showed that Kinect is useful in elderly fall prevention and exercising with it improves the results of balance assessment scales in the experimental group. Stone and Skubic [59] used Kinect to study gait in 5 elderly subjects in their home during a 4-month period and proposed a methodology for gait monitoring using Kinect. These studies show that Kinect has been an acceptable tool in elderly monitoring and exercise.

Computerized rehabilitation of other physical/cognitive disabilities has been tried using Kinect. For example, Han et al. [60] evaluated usability of Kinect in evaluating the reachable workspace in 22 patients with facioscapulohumeral muscle dystrophy. They showed that Kinect's measurements were in accordance with the clinical observation. Galna et al. [61] used Kinect for retraining functions in patients with Parkinson's disease (PD). They evaluated their rehab system with 9 patients with PD and concluded that Kinect-based games are, in general, safe and feasible for PD rehabilitation but interventions should be carefully reviewed for safety and efficacy in the home. Also, Pompeu et al. [62] showed that 7 patients with PD improved their* g*-minute walk test after playing Kinect-based games for 14 hours during 4 1/2 weeks. Ulaşli et al. [63] examined the utility of Kinect in training patients with leukodystrophy. Only one patient participated who demonstrated improvements in functional independency, mobility, walking speed, and balance as measured by standard quantitative assessments. Parry et al. [64] proposed video gaming for physical rehabilitation after burn injury; they compared Kinect versus PlayStation 3 and showed that subjects who played with the Kinect achieved significantly greater ROMs in shoulder flexion, shoulder abduction, and elbow flexion. González-Ortega et al. [65] showed that Kinect may be useful in monitoring and rehabilitation of individuals with body scheme dysfunctions and left-right confusion. Ortiz-Gutiérrez et al. [66] showed that in 25 patients with multiple sclerosis (MS) who received a Kinect-based intervention visual preference and the vestibular balance improved significantly greater than the 25 control MS patients who received conventional treatment. Two studies on cerebral palsy (CP) were found; Luna-Oliva et al. [67] used Kinect for rehabilitation of 11 children with CP in an 8-week study and they showed significant improvements in the standard motor assessments; the improvements were still present in the follow-up examination. Chang et al. [68] used Kinect for CP rehabilitation; they showed that 2 adolescents with CP demonstrated high motivations for exercising with Kinect and improved their performance during the intervention. Ilg et al. [69] used Kinect in home setting of children with ataxia in an 8-week training experiment and showed that their ataxia symptoms were significantly reduced. Holmes et al. [70] investigated the utility of Kinect in high intensity exercise for patients with cystic fibrosis and concluded that it may be a suitable alternative for conventional exercise. These studies show that Kinect has impacted various fields of RE and engineers and clinicians have shown increasing interest in using it as a module in rehab systems.

### 5.2. Systems Evaluated by Healthy Subjects

The convenience and affordability of the Kinect sensor with its acceptable accuracy invited a lot of interest among RE developers to propose stroke rehab frameworks based on Kinect. Table 2 shows some of many studies that have been published in the past 2-3 years since Kinect has been available to the market (2010).

Some of these studies proposed a platform based on Kinect to get more data out of or quantify a clinical test. For example, Hsiao et al. [71] recorded the 3D position of the upper limb joint in the Box and Block Test (or BBT) to provide further data besides a simple BBT score.

In motion capture for stroke rehabilitation, Kinect has been used to measure balance and upper and lower extremities motions. Gonzalez et al. [72, 73] used Kinect to estimate the center of mass (CoM) in balance analysis. In [74–80], the authors proposed different systems for upper limb rehabilitation. Saini et al. [81], Yeh et al. [82], Borghese et al. [83], and Kitsunezaki et al. [84] proposed systems which promoted rehabilitation of both upper and lower extremities. Kinect has also been used for monitoring fine motions such as finger movements; Cordella et al. [85] developed a system for individual finger tracking using Kinect.

The Kinect's application has not been limited to stroke rehabilitation and has extended to other therapeutic fields; for example, Lozano-Quilis et al. [86] used the Kinect sensor for analyzing movement of MS patients and Abdur Rahman et al. [87] used Kinect in a multimedia interactive therapy system for disabled children. Also, Cervantes et al. [88] developed a cognitive rehab system using Kinect.

Sensor fusion, with Kinect as one of the sensors, has been tried by Chavezguevara et al. [89], Sadihov et al. [90], and Hondori et al. [40]. Kinect was combined with haptic devices and sensors in [89, 90] to improve user's experience in VR. In [40], inertial sensors were used to make sensor-enabled utensils to capture fine motions while Kinect was recording gross movements during eating as a bimanual task involving both affected and unaffected upper limbs. The discussed studies in this section did little or no clinical evaluation of their systems. They are summarized in Table 3 where notable properties of them are also itemized.

## 6. Usability of Kinect-Like Sensors in PT and Rehabilitation

**Figure 3:**
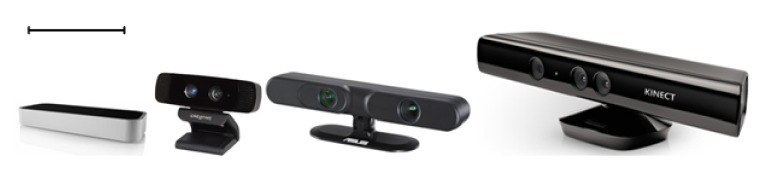
Different motion sensing devices roughly scaled to compare their sizes: from left to right Leap Motion Controller, Intel Creative Gesture Camera, Asus Xtion, and Microsoft Kinect; the scale bar is 10 cm.

In this section we discuss three other commercially available and affordable sensors whose impact on RE may be similar to Kinect. Since they are relatively newer, there are only few clinical studies that used them; we will refer to these studies in the current section. These sensors are shown in Figure 3 and from right to left they are Microsoft Kinect, Xtion Pro Live, Intel-Creative camera, and Leap Motion controller. Note that the devices' specifications are subject to change and the information provided in the section is true at the time of preparing this review article.

### 6.1. Leap Motion Controller

Leap is another motion sensing device by Leap Motion. The main motivation behind building Leap was to alleviate 3D modeling which used to be accomplished using conventional human computer input devices such as mouse and keyboard. Leap Motion has partnered with both Asus and Hewlett Packard to embed its technology within future Asus/HP notebook/PCs.

The leap unit is a 3′′ × 1.2′′ × 0.5′′ USB peripheral device. It is designed to be positioned in front of the screen (of a notebook or PC) on the table. The device consists of 3 infrared LEDs and 2 cameras. Leap's field of view is a hemisphere above the device with radius between 25 and 600 millimeters. The leap detects and tracks both fingers and tools (with similar shape of fingers such as pen). It provides developers with hand and fingers information such as fingertip position, hand velocity, and hand/finger direction. As stated in Leap Motion's webpage, the skeletal model of the hand will be released in the near future. But Leap Motion has not announced any decision on giving developers access to raw data (by the date of this paper). In terms of gesture recognition, so far Leap's SDK provided four gestures of key tap, screen tap, swipe, and circle. Leap's SDK is available for Windows, Linux, and OSX platforms and programming languages of C++, C#, Unity, Java, Javascript, and Python. Examples of clinical studies that have used Leap Motion controller are [91, 92].

### 6.2. Intel Creative Camera

Creative Interactive Gesture Camera by Intel is a 4.27′′ × 2.03′′ × 2.11′′ depth sensor. It can be plugged in via USB, tracking close range interaction. The device consists of an HD webcam, depth sensor, and dual array microphone. Its depth sensing range is 0.15–0.40 m (6–15.7 inch). Examples of clinical studies that have used the Intel-Creative camera are [93, 94].

### 6.3. ASUS XTION Camera

Asus introduced Xtion to the motion capture market in 2012. The company released different versions of Xtion for game as well as development purposes (such as Xtion Pro Live). Xtion uses an infrared sensor and adaptive depth detection to track precise body movements. Xtion Pro Live can detect/track whole body as well as hand/gestures. Xtion offers more than 8 predefined poses such as push, click, circle, and wave. It benefits from a plug and play USB design and OPNI NITE as a development middleware.

### 6.4. Comparison between the Sensors

Table 4 compares the discussed depth sensor's specifications in more detail. We recommend developers to select their depth sensor according to the requirements of their problem. For example, when finger individuation is the focus of the rehab training, the Leap sensor could be the best option. For tabletop applications where exercising upper extremity is desired, Intel Creative camera offers adequate depth sensing range. In lower limb or whole-body motion tracking, Kinect or Asus Xtion is preferable.

## 7. Summary and Conclusion

This paper reviewed the literature on Kinect-based rehabilitation. We first reviewed similar systems before Kinect was introduced to shed light on the later impact of Kinect; we reviewed limitations and possible errors in those methods. Besides lack of fidelity in the motion capture, these systems were only able to track one or a few points of the human body (e.g., palm of the hand, face, etc.). Because of computational load required to extract human skeleton from an RGB image, building a real time interactive system using contrast-based imaging is not very reliable. In contrast, depth imaging devices such as Kinect are preferable to developers because they provide a Software Development Kit (SDK) which gives access to skeletal tracking data and can be used directly in rehabilitation game developments.

Since the arrival of Microsoft Kinect, many rehabilitation engineers have employed Kinect in their systems. Although Kinect-based motion capture is far more accurate than RGB systems, it comes with it its own limitations and errors. We reviewed studies which evaluated Kinect's accuracy and robustness with more accurate systems such as optoelectronic systems. Their results showed that Kinect can be an acceptable tool for rehabilitation due to its low cost and adequate accuracy. However, developers should consider issues such as occlusion and noise in skeleton tracking. These problems can partly be solved by applying Kalman filtering, sensor fusion, and calibration.

Subsequently, a broad range of rehabilitation systems which used Kinect were discussed. We investigated systems with and without clinical evaluation. These studies targeted upper and/or lower limb rehabilitation, balance monitoring/training, and range of motion exercises among other physical and cognitive tasks and showed that the patients and therapists accepted the Kinect-based rehab systems. In some cases, patients showed significant improvements on their clinical assessments (Fugl-Meyer score Box and Block Test, 6-minute walking tests, etc.). Although other studies proved their frameworks, game engines, and so forth, lacking clinical evaluation invites questions on the practical effectiveness of the method/systems.

In comparison with Kinect, we also compared three other commercially available depth sensors, namely, Leap Motion, Intel Creative camera, and Asus Xtion. The specifications of these devises were discussed in detail. This aims to help rehabilitation system developers select their depth sensor according to the requirements of the problem. For example, for hand tracking with individual fingers the Leap sensor is a more suitable option than the Kinect and for close range tracking Intel Creative camera performs better than Kinect, but when full body motion is required, Kinect or Asus Xtion is appropriate.

## Figures and Tables

**Table 1:** Different types of motion capture system.

Nonoptoelectronics MoCap	Optoelectronics MoCap
(i) Inertial sensors (ii) Magnetic systems (iii) Wearable systems (iv) Mechanical systems	(i) Marker trigonometry with IR cameras (ii) Contrast-based (a) With color markers (b) With skin detection (iii) Depth-based

**Table 2:** Clinically evaluated systems using Kinect.

Article	Targeted disability	Study type	Purpose	Evaluation	Conclusion
Pastor et al. [50]	Stroke	Rehab	Upper limb	One patient (10 days)	(i) Potential for further investigation

Chang et al. [47]	Stroke	Rehab	Upper limb	Four patients (2 weeks)	(i) Helped with preservice training (ii) Increased independence (iii) Helped with community integration

Wiederhold and Riva [46]	Stroke	Rehab	Lower limb	15 patients (20 sessions; each 45 min, 3–5 sessions per week)	(i) Framework was evaluated by patients (ii) Within subject assessment shows significant improvement in balance recovery

Exell et al. [48]	Stroke	Rehab	Upper limb	One patient (18 sessions)	(i) Mean joint angle error reduced from 35 to 51% across 3 joints

Gama et al. [49]	Elderly	Rehab	Upper limb	Three elderly subjects (1 session)	(i) Kinect proved accurate (ii) Potential for further investigation

Acosta [51]	Stroke	Rehab	Upper limb	Six patients + 5 controls in a 10-day study	(i) Patients liked the game (ii) Clinical scores were maintained or improved

Adams et al. [52]	Stroke	Assessment	Upper limb	14 hemiparetic patients in Virtual Occupational Therapy Assistant (VOTA)	(i) Moderate correlation between VOTA derived metrics and WMFT scores

Bao et al. [55]	Stroke	Rehab	Upper limb	Five patients in a 3-week study	(i) FM score and WMFT scores improved (ii) Improvements maintained in a 12-week follow-up (iii) fMRI showed that learning the neurological underpinning of learning a task using Kinect may differ from healthy controls

Lee [53]	Stroke	Rehab	Upper limb	14 patients	(i) Control and study patients both showed improvements (ii) Experimental group who received Kinect treatment demonstrated more changes from pre- to posttreatment

Sin and Lee [54]	Stroke	Rehab	Upper limb	40 patients	(i) Experimental group (*n* = 20) who received Kinect treatment showed significantly greater improvements in FM and BBT score than the control patients (*n* = 20)

Stone and Skubic [59]	Elderly	Monitoring	Gait	Four older adults in a 4-month home-based study	(i) They proposed a methodology for gait monitoring using Kinect

Pu et al. [57]	Elderly	Monitoring	Balance and gait	100 older adults	(i) Investigated key factors in the balance of older adults (ii) Showed that the static and dynamic balance functions were explained with distinct factors

Dutta et al. [56]	Elderly	Assessment	Balance	10 older adults	(i) They showed that the maximum Center-of-Mass (CoM)-Center-of-Pressure (CoP) lean-angle correlates significantly with the clinical balance scores (i.e., Berg Balance Scale)

Galna et al. [61]	Parkinson's disease	Rehab	Gait	Nine patients	(i) Use of Kinect is safe and feasible for PD rehabilitation (ii) Interventions should be carefully reviewed for safety and efficacy in the home

Pompeu et al. [62] showed 7 patients with PD	Parkinson's disease	Rehab gait		Seven patients in a 14-hour study (during 4 (1/2) weeks)	(i) Patients improved their 6-minute walk test after

Luna-Oliva et al. [67]	Cerebral palsy	Rehab	ADL	11 children with CP in an 8-week study	(i) Significant improvements in the standard motor assessments (ii) The improvements were still present in the follow-up examination

Chang et al. [68]	Cerebral palsy	Rehab	ADL	Two children with CP	(i) Patients demonstrated high motivations for exercising with Kinect (ii) They improved their performance during the intervention

Ilg et al. [69]	Children with ataxia	Rehab		Eight-week training	(i) Ataxia symptoms were significantly reduced

Holmes et al. [70]	Cystic fibrosis	High intensity exercise	NA	10 patients	(i) It may be a suitable alternative for conventional exercise

Ortiz-Gutiérrez et al. [66]	Multiple sclerosis	Rehab	Vision and balance	50 patients	(i) Study group (*n* = 25) showed significant improvement in visual preference and the vestibular balance, more significantly than control patients (*n* = 25) who received conventional treatment

Han et al. [60]	Facioscapulohumeral muscle dystrophy	Assessment		22 patients	(i) Kinect's measurements were in accordance with clinical observations

Parry et al. [64]	Burn injury	Rehab	Range of motion	30 children	(i) Subjects who played with the Kinect achieved significantly greater ROMs in shoulder flexion, shoulder abduction, and elbow flexion than the control group who received treatment via PlayStation 3

Ulaşli et al. [63]	Leukodystrophy	Rehab	Balance and gait	One patient	(i) Subject demonstrated improvements in functional independency, mobility, walking speed, and balance as measured by standard quantitative assessments

**Table 3:** Nonclinically evaluated systems using Kinect.

Article	Summary of findings
Lozano-Quilis et al. [86]	Provided MS patients with motor rehab exercises using Kinect

Gonzalez et al. [72]	Estimated CoM in human subjects using Kinect data in real time

González et al. [73]	Compared CoM estimation for in-home rehab using Kinect + Wii vs. Vicon

Hsiao et al. [71]	Developed digitized Box and Block Test to measure unilateral gross manual dexterity

Chavezguevara et al. [89]	Provided therapists a controller to operate the exoskeleton based on force feedback and limb's position retrieval

Sadihov et al. [90]	Enhanced immersion and providing sensory feedback in VR environment rehab training using motion-based tactile rendering algorithm

Pogrzeba et al. [95]	Provided motion analysis system

Cordella et al. [85]	Provided marker-based finger tracking with Bayesian estimation

Cervantes et al. [88]	Conducted a case study for cognitive rehab

Abdur Rahman et al. [87]	Provided multimedia (Second Life) interactive therapy for disabled children

Gotsis et al. [74]	Created a platform for prototyping of VR-based games for rehab

Calin et al. [75]	Monitored patients using Kinect

Saini et al. [81]	Proposed a framework for gamified rehab

Yeh et al. [82]	Proposed an interactive interface for games in stroke rehab

Borghese et al. [83]	Integrated Kinect with a fully adaptive game engine for stroke rehab

Brokaw and Brewer [76]	Developed HAMSTER: a Kinect-based home rehab system

Huang et al. [77]	Integrated Kinect and Smart Glove into a hand motion capture system

Gama et al. [49], Da Gama et al. [78]	Developed a system to provide guidance and correction in therapeutic exercises

de Urturi et al. [79]	Developed JeWheels: an exergame to improve motor skills and cognitive abilities for wheelchair users

Kitsunezaki et al. [84]	Developed a system for real time ROM measurement in standard walking tests

Scherer et al. [80]	Enhanced functional brain mapping by tracking self-paced hand opening and closing

Yao et al. [96]	Propose Kinect as assistance for therapists to improve the treatment process and increase patients' motivation

Galeano et al. [97]	Proposed a balance training tool using Kinect and Wii

Borghese et al. [98]	Investigated the needs of the patients and clinicians in a home-based rehabilitation scenario and identified Kinect as one of the main tools for such systems

Cipresso et al. [99]	Targeted unilateral spatial neglect which is in patients with stroke and analyzed different grasping tasks to evaluate the patient's ability in handling virtual objects in both sides of their workspace in an ecological way

Brokaw et al. [100]	Used Kinect to detect and limit compensatory postures in robotic rehabilitation

Venugopalan et al. [101]	Proposed a home-based system for assessment and rehab of patients with traumatic brain injury and validated it with 2 healthy individuals

Gibson et al. [102]	Evaluate the feasibility of using theKinect for activity classification and behavioral mapping of patients at bed rest

Metcalf et al. [103]	Used Kinect's depth imaging and established a finger joint measurement method that is more accurate than clinically based alternatives and manual measurement methods

Guerrero and Uribe-Quevedo [104]	Developed a software that tracks patient's posture which also guides the patient to match their posture with a model posture

Lange et al. [105]	Developed an interactive game-based rehabilitation tool using the Kinect to improve balance function in patients with neurological injury

**Table 4:** Comparison of the four discussed depth sensors.

Feature	Kinect (1st generation)	Leap	Creative	Xtion Pro Live
Size	14.8′′ × 5.9′′ × 4.8′′	3′′ × 1.2′′ × 0.5′′	4.27′′ × 2.03′′ × 2.11′′	7′′ × 1.4′′ × 2′′

Frame rate (fps)	9–30	30	30	30/60

Maximum depth resolution	640 × 320	N.A.	QVGA (320 × 240)	VGA (640 × 480) with 30 fps QVGA (320 × 240) with 60 fps

Maximum RGB resolution	640 × 320	N.A.	1280 × 720	SXGA (1280 ∗ 1024)

Access to raw image	Yes	No	Yes	Yes

Depth sensing range	Seated mode: physical limits 0.4–3 m Sweet spot: 0.8–2.5 m Default: physical limits: 0.8–4 m Sweet spot: 1.2–3.5 m	0.025 to 0.6 m	0.15–0.4 m	0.8–3.5 m

Diagonal field of view	27° U/D 43.5° V 57.5° H	N.A.	73°	70° D 45° V 58° H

Compatible platform	Win 7, 8	Win 7, 8 Ubuntu Linux Mac OS	Win 7	Win XP, Vista, 7 Linux Ubuntu 10.10 Android (by request)

Programming language	C++, C# Visual Basic	C++, C# JAVA Python Javascript Objective-C Mono Unity, Unity	C++, C#, JAVA	C++/C#, JAVA

Tracking	Whole body	Hand/finger/tool	Hand/object	Whole body/hand
